# Mesoporous polydopamine delivery system for intelligent drug release and photothermal-enhanced chemodynamic therapy using MnO_2_ as gatekeeper

**DOI:** 10.1093/rb/rbad087

**Published:** 2023-09-22

**Authors:** Zhaoyang Wang, Zekai Li, Yuehua Shi, Leyong Zeng

**Affiliations:** Key Laboratory of Medicinal Chemistry and Molecular Diagnosis of the Ministry of Education, Chemical Biology Key Laboratory of Hebei Province, State Key Laboratory of New Pharmaceutical Preparations and Excipients, College of Chemistry and Materials Science, Hebei University, Baoding 071002, P.R. China; Key Laboratory of Medicinal Chemistry and Molecular Diagnosis of the Ministry of Education, Chemical Biology Key Laboratory of Hebei Province, State Key Laboratory of New Pharmaceutical Preparations and Excipients, College of Chemistry and Materials Science, Hebei University, Baoding 071002, P.R. China; Key Laboratory of Medicinal Chemistry and Molecular Diagnosis of the Ministry of Education, Chemical Biology Key Laboratory of Hebei Province, State Key Laboratory of New Pharmaceutical Preparations and Excipients, College of Chemistry and Materials Science, Hebei University, Baoding 071002, P.R. China; Key Laboratory of Medicinal Chemistry and Molecular Diagnosis of the Ministry of Education, Chemical Biology Key Laboratory of Hebei Province, State Key Laboratory of New Pharmaceutical Preparations and Excipients, College of Chemistry and Materials Science, Hebei University, Baoding 071002, P.R. China

**Keywords:** mesoporous polydopamine, MnO_2_ gatekeeper, chemotherapy, photothermal therapy, chemodynamic therapy

## Abstract

The non-specific leakage of drugs from nanocarriers seriously weakened the safety and efficacy of chemotherapy, and it was very critical of constructing tumor microenvironment (TME)-responsive delivery nanocarriers, achieving the modulation release of drugs. Herein, using manganese dioxide (MnO_2_) as gatekeeper, an intelligent nanoplatform based on mesoporous polydopamine (MPDA) was developed to deliver doxorubicin (DOX), by which the DOX release was precisely controlled, and simultaneously the photothermal therapy (PTT) and chemodynamic therapy (CDT) were realized. In normal physiological environment, the stable MnO_2_ shell effectively avoided the leakage of DOX. However, in TME, the overexpressed glutathione (GSH) degraded MnO_2_ shell, which caused the DOX release. Moreover, the photothermal effect of MPDA and the Fenton-like reaction of the generated Mn^2+^ further accelerated the cell death. Thus, the developed MPDA-DOX@MnO_2_ nanoplatform can intelligently modulate the release of DOX, and the combined CDT/PTT/chemotherapy possessed high-safety and high-efficacy against tumors.

## Graphical Abstract

**Figure:**
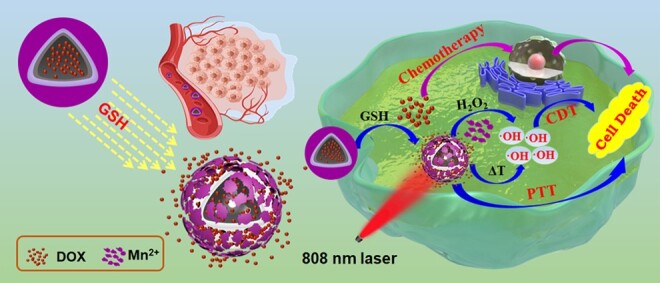

## Introduction

Cancers possessed high mutation rate and metastasis, and caused great threatening to the life of human beings [1–3]. In clinical, chemical drugs were widely applied, but conventional chemotherapy still faced with great challenges. Especially, many patients were seriously suffering from the side effects of chemotherapy, and the drug resistance was also a key factor of leading to the failure of chemotherapy [4, 5]. Therefore, it was very urgent of developing new chemotherapy drugs with high safety and high efficacy.

In the past decades, functional nanomaterials were investigated as drug carrier, which provided new strategies for improving chemotherapy performance [6, 7]. By constructing delivery nanosystems, the stability of drugs was improved, and the drug accumulation at tumor sites was significantly increased [8, 9]. Different nanocarrier systems have been investigated, including metal-organic frameworks [10, 11], liposomes [12, 13], inorganic oxide nanoparticles [14, 15], graphene and mesoporous carbon nanospheres [16–18]. Owing to the good photothermal performance and rich channels, Prussian blue and polydopamine with mesoporous structure (MPDA) had received much attention as nanocarriers [19–21]. Especially, the abundant aromatic rings of MPDA made it possible to load chemical drugs by electrostatic adsorption, π–π stacking, and hydrophobic–hydrophobic interactions [22, 23]. However, before reaching the tumor sites, the premature leakage of drugs could reduce the therapeutic efficacy, and also bring toxicity to normal tissues [24]. Therefore, it was very important of constructing intelligent delivery nanoplatform, avoiding the non-specific leakage of drugs in normal physiological environment, but achieving the controllable release of drugs in tumor microenvironment (TME).

Recently, exogenous/endogenous-responsive delivery nanoplatforms were reported to realize the modulation release of drugs. When they were delivered into tumor sites, the protective layer can be destroyed, which accelerated the release of drugs [25, 26]. TME-responsive liposomes [27, 28], polypeptides [29] and inorganic nanomaterials with disulfide bond [30] could cleave into small nanocomponents by endogenous substances, by which the drugs release was caused. Thermosensitive polymer, a kind of temperature-responsive phase-transition material, can transition from solid into liquid under the triggering of photo-thermal conversion, by which the accelerated drug release and photothermal therapy (PTT) were realized [31, 32]. It was well known that glutathione (GSH) and hydrogen peroxide (H_2_O_2_) with high-concentration were present in TME, and it was very meaningful of designing TME-responsive nanoplatform to modulate the release of drugs [33–35]. Importantly, based on the Fenton/Fenton-like reactions of metal ions, TME-responsive chemodynamic therapy (CDT) showed promising application in cancer treatment [36–38]. Among many TME-responsive nanoplatforms, manganese dioxide (MnO_2_) was considered as the most promising protective layer, owing to its GSH-responsive degradation characteristics [39, 40]. Moreover, the reduced Mn^2+^ can convert endogenous H_2_O_2_ to highly toxic hydroxyl radical (·OH) for CDT [41–44]. Therefore, using MnO_2_ as protective layer, the release of drugs can effectively be modulated, and simultaneously the CDT amplified the anti-tumor efficacy.

**Scheme 1.**
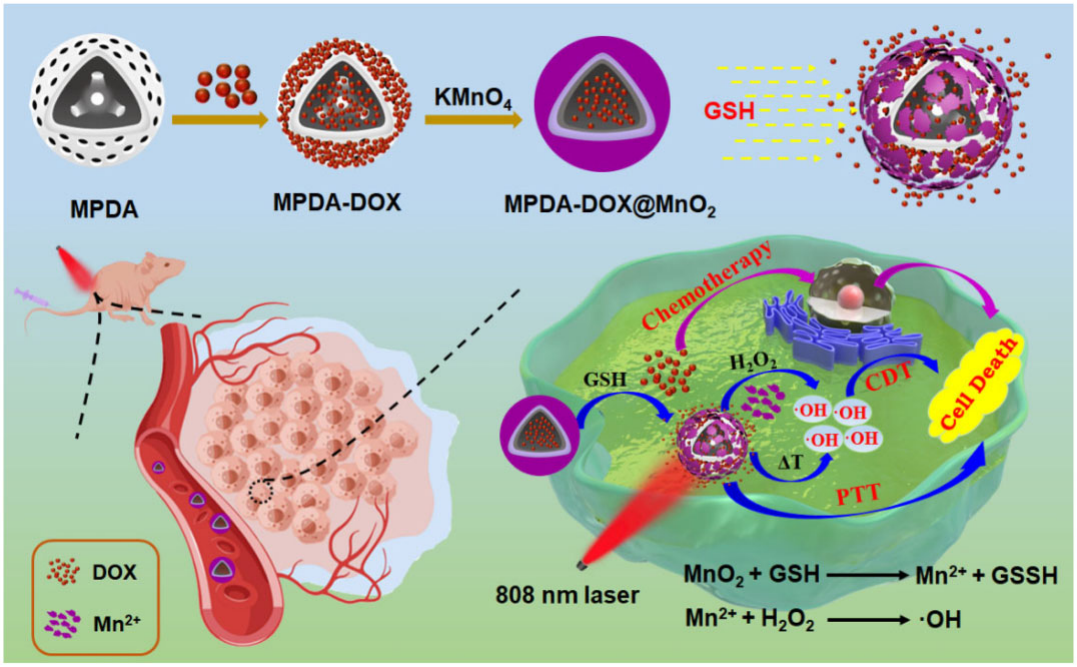
Schematic diagram of MPDA-DOX@MnO_2_ for intelligent drug release and photothermal-enhanced CDT.

In this work, by designing MnO_2_ as ‘gatekeeper’, an intelligent delivery nanoplatform (MPDA-DOX@MnO_2_) was constructed for simultaneous CDT/PTT/chemotherapy, in which MPDA nanoparticles were used as nanocarrier to load doxorubicin (DOX), and GSH-responsive MnO_2_ was used as protective player to modulate the DOX release. In Scheme 1, MPDA nanospheres were firstly prepared, and DOX was loaded onto MPDA (MPDA-DOX) using electrostatic adsorption. Finally, MnO_2_ shell was *in situ* grown on MPDA-DOX to form MPDA-DOX@MnO_2_. In normal physiological environment, the stable MnO_2_ shell avoided the premature release of DOX. However, in TME, the overexpressed GSH reduced MnO_2_ to generate Mn^2+^, which led to the degradation of MnO_2_ shell and allowed the release of DOX. Furthermore, the photothermal property of MPDA accelerated the ·OH generation of Mn^2+^. Thus, the MPDA-DOX@MnO_2_ nanoplatform effectively modulated the DOX release, and achieved the combination of chemotherapy/PTT/CDT, opening new avenues for the development of intelligent delivery nanoplatforms.

## Experimental section

### Reagents

Dopamine hydrochloride (DA·HCl), 1,3,5-Trimethylbenzene (TMB, 97%), pluronic F-127 (F-127), ammonia solution (NH_3_·H_2_O, 25%), methoxypolyethylene glycol amine (mPEG-NH_2_, MW = 2000), doxorubicin hydrochloride (DOX), methylene blue (MB), tris(hydroxymethyl)aminomethane (Tris, 99.9%), H_2_O_2_ (30%), glutathione (GSH) and 5,5’-dithiobis(2-nitrobenzoicacid) (DTNB) were all purchased from Aladdin (Shanghai, China). Methyl thiazolyl tetrazolium (MTT), phosphate buffered saline (PBS), DMEM culture medium, antibiotic/antimycotic solution, fetal bovine serum (FBS), 2,7-dichlorofluorescein diacetate (DCFH-DA), Hoechst, Calcein-AM/PI (CAM/PI) staining, and GSH assay kit were purchased from Solarbio (Beijing, China).

### Preparation of MPDA-DOX@MnO_2_

#### Synthesis of MPDA-DOX

Firstly, MPDA nanoparticles were prepared [45]. Five milliliters of DOX at different concentrations (0, 200, 300, 400, 500, 600, 700, 800, 900 and 1000 μg/ml) were mixed with 5 ml of MPDA (1 mg/ml). Then, they were stirred for 24 h. Finally, the MPDA-DOX nanocomposites were centrifuged and dispersed in 5 ml of deionized water.

#### Synthesis of MPDA-DOX@MnO_2_

One hundred and twenty microliters of KMnO_4_ (2 mg/ml) was added into 2 ml of MPDA-DOX (100 μg/ml). Then, 240 μl of formamide was dropped. After 15 min, MPDA-DOX@MnO_2_ nanoparticles were obtained. Finally, they were centrifuged, and dispersed in 2 ml of deionized water. Alternatively, by adding 120 μl of KMnO_4_ at different concentrations (0.7, 1.0, 1.3, 1.7, 2.0 and 2.3 mg/ml) and 240 μl of formamide into 2 ml of MPDA (100 μg/ml), MPDA@MnO_2_ nanospheres were also prepared.

### Characterization

The morphological changes of the nanomaterials were observed with a Tecnai F30 transmission electron microscope (FEI, USA) and a Nova NanoSEM450 scanning electron microscope (FEI, USA). The size and potentials were recorded on a Nano-ZS Zetasizer (Malvern, UK). The Brunauer–Emmett–Teller (BET) surface area and pore size distribution were measured using an ASAP2020M automatic specific surface area and t porosity analyzer (Micromeritics, USA). A K-α X-ray photoelectron spectrometer (Thermo Fisher, USA) was used to analyze the X-ray photoelectron spectroscopy (XPS). A Lambda 365 ultraviolet–visible (UV–vis) spectrophotometer (PerkinElmer, Korea) and a Nicolet iS10 Fourier transform infrared (FT-IR) spectrometer (Thermo Fisher, USA) were used to measure the UV–vis absorption and FT-IR spectra.

### DOX loading and release

#### DOX loading

Five milliliters of DOX with different concentrations (0.2, 0.4, 0.6, 0.8, 1.0, 1.2, 1.4, 1.6, 1.8 and 2.0 mg/ml) were added to 5 ml of MPDA (1 mg/ml), and stirred at room temperature in the dark for 24 h. By centrifugation, the MPDA-DOX was obtained, and the absorbance of the obtained supernatants was recorded. In addition, by measuring the absorbance of free DOX, the standard curve was drawn and the DOX loading was calculated.

#### DOX release

Three milliliters of MPDA-DOX and MPDA-DOX@MnO_2_ (5 mg/ml) in dialysis bag were placed in 57 ml of PBS (pH = 7.4, 6.5 and 5.5). At different time points, 1 ml of PBS was removed, and 1 ml of newly PBS was added. Finally, the absorbance of removed PBS was measured, and the cumulative release of DOX was calculated. Alternatively, by adding GSH (2.5, 5.0, 7.5 and 10 mM) in PBS (pH = 5.5), the cumulative DOX release in MPDA-DOX@MnO_2_ was also measured.

### Photothermal performance

#### Photothermal conversion

An infrared thermal imager was employed to detect the temperature. Briefly, 1 ml of MPDA-DOX@MnO_2_ (0, 30, 60, 90, 180, 240 and 300 μg/ml) was irradiated by an 808 nm laser (1.0 W/cm^2^) for 10 min, and the temperature was recorded. In addition, by varying the power density (0.3, 0.6, 0.9, 1.2 and 1.5 W/cm^2^), the temperature change of MPDA-DOX@MnO_2_ (180 μg/ml) was also monitored.

#### Photothermal stability

One milliliter of MPDA-DOX@MnO_2_ (180 μg/ml) was irradiated (1.0 W/cm^2^) for 10 min. Then, the laser was turned off, and the temperature was recorded. Finally, the photothermal conversion efficiency was calculated. Moreover, by repeating on/off laser for five times, the photothermal stability was measured.

### GSH consumption and ·OH generation

#### GSH consumption

Two milliliters of MPDA@MnO_2_ (0, 30, 60, 90, 120 and 180 μg/ml) (pH = 6.5) was incubated with 40 μl of GSH (100 mM). After 30 min, 200 μl of supernatant was mixed with 800 μl of DTNB (0.1 mM). Finally, the absorbance of 2-nitro-5-thiobenzoic acid (TNB) was measured. In addition, by varying the water bath temperature (25°C, 37°C and 49°C), the GSH depletion was also recorded.

#### ·OH generation

Two milliliters of MPDA@MnO_2_ (150 μg/ml) (pH = 6.5) containing NaHCO_3_ (25 mM) was mixed with 40 μl of GSH (10, 20, 30, 40, 50, 100, 200, 300, 400, 500 mM). After 2 h, 1 ml of supernatant was incubated with 100 μl of MB (100 μg/ml) containing of H_2_O_2_ (600 μM) for 30 min. Finally, the absorbance of MB was recorded. Moreover, by varying temperatures (25°C, 37°C and 49°C), incubation time (0–30 min) and H_2_O_2_ concentration (100, 200, 300, 400, 500 and 600 μM), the absorbance was also recorded.

### *In vitro* biocompatibility and cellular ·OH generation

#### Cytotoxicity

MDA-MB-435 cells were cultured in a 96-well plate (1 × 10^4^ cells/well) for 24 h, and incubated with 100 μl of MPDA and MPDA@MnO_2_ (0, 30, 60, 90, 120, 150 and 180 μg/ml). After 20 h, the survival rate was recorded by a Synergy HTX multimode microplate reader (Biotek, USA).

#### Cellular uptake

One milliliter of MPDA-DOX and MPDA-DOX@MnO_2_ (90 μg/ml) was incubated with MDA-MB-435 cells for 2, 4 and 6 h. Then, they were fixed, and stained with Hoechst. Finally, the cells were observed on an LSM-880 laser scanning confocal microscope (Zeiss, Germany).

#### Cellular ·OH generation

One milliliter of MPDA@MnO_2_ (150 μg/ml) containing H_2_O_2_ (100 μM) was incubated with cells for 4 h. Then, 1 ml of medium containing DCFH-DA (10 μM) was added and incubated for 30 min. Finally, the cells were stained with Hoechst, and the fluorescence of 2,7-dichlorofluorescein (DCF) was observed. Alternatively, the cells were irradiated (1.0 W/cm^2^) for 5 min, and the DCF fluorescence was also observed.

### *In vitro* CDT/PTT/chemotherapy

The cells were incubated with 200 μl of MPDA@MnO_2_ (0, 30, 60, 90, 120, 150 and 180 μg/ml) (pH = 7.4) for 4 h. Then, they were irradiated at different power densities (0, 0.75 and 1.5 W/cm^2^) for 5 min. After 20 h, the viability was recorded to evaluate the *in vitro* PTT performance. Moreover, 200 μl of MPDA-DOX and MPDA-DOX@MnO_2_ (180 μg/ml) (pH = 7.4) was incubated with MDA-MB-435 cells for different time (0, 6, 12, 18, 24 and 48 h), and the viability was also measured to evaluate the *in vitro* chemotherapy performance.

With the presence of H_2_O_2_ (100 μM), 200 μl of MPDA-DOX@MnO_2_ at different concentrations (0, 30, 60, 90, 120, 150 and 180 μg/ml) (pH = 6.5) was incubated with MDA-MB-435 cells for 20 h, and the cell viability was recorded. Alternatively, after 4 h incubation, the cells were irradiated (1.5 W/cm^2^) for 5 min, and the *in vitro* CDT/PTT/chemotherapy performance was tested. In addition, the cells were incubated with MPDA@MnO_2_ and MPDA-DOX@MnO_2_ with/without irradiation (1.5 W/cm^2^) for 5 min. After 20 h, the cells were stained with CAM/PI, and the live/dead cells were observed.

### *In vivo* biocompatibility and CDT/PTT/chemotherapy

The animal experiments were done in Experiment Animal Center of Hebei University (SYXK (Ji) 2022-009), and the protocols were approved by Experimental Animal Welfare Ethics Committee of Hebei University.

#### *In vivo* biocompatibility

Healthy nude mice were injected intravenously with 100 μl of saline and MPDA-DOX@MnO_2_ (500 μg/ml). After 3 weeks, the mice were executed, and the blood indexes were analyzed. Moreover, the major organs were collected to perform H&E staining.

#### *In vivo* CDT/PTT/chemotherapy

The *in vivo* experiment included seven groups: Saline, Saline (L+), MPDA-DOX, MPDA@MnO_2_, MPDA-DOX@MnO_2_, MPDA@MnO_2_ (L+), and MPDA-DOX@MnO_2_ (L+). Briefly, 100 μl of different samples (500 μg/ml) was injected through the tail vein, respectively. After 24 h, the tumors of mice in laser groups were irradiated (1.0 W/cm^2^) for 5 min, and the temperature change was detected. By measuring the body weight and tumor volume, the *in vivo* CDT/PTT/chemotherapy was evaluated, and the survival rates were also recorded. In addition, the main organs in the groups of Saline and MPDA-DOX@MnO_2_ (L+) were removed for H&E staining.

## Results and discussion

### Characterization of MPDA-DOX@MnO_2_

**Figure 1.**
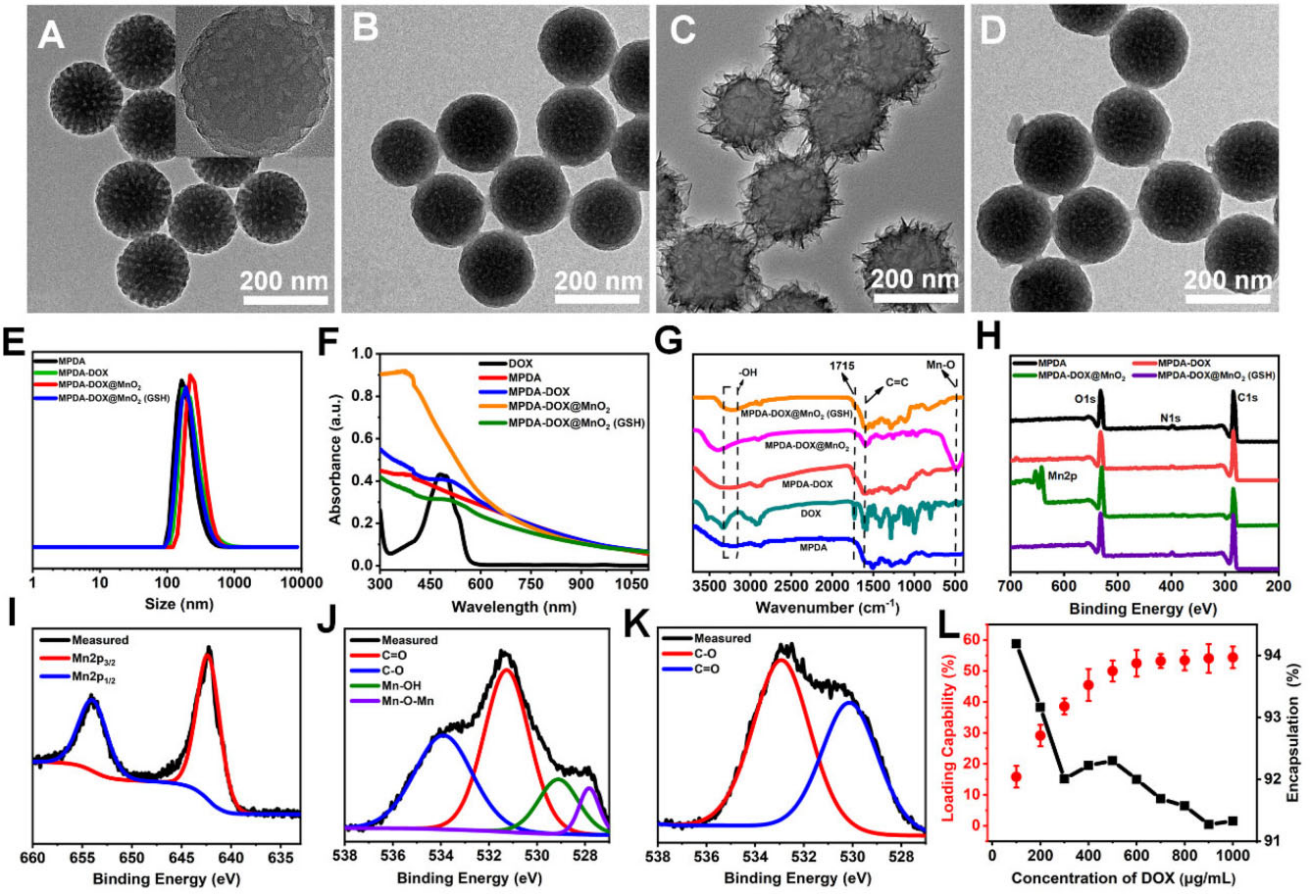
(**A–D**) TEM images of MPDA, MPDA-DOX, MPDA-DOX@MnO_2_ and MPDA-DOX@MnO_2_ (GSH). (**E**) DLS curves. (**F**) UV–vis absorption spectra. (**G**) FT-IR spectra. (**H**) XPS spectra. (**I**) Mn2p and (**J**) O1s XPS spectra of MPDA-DOX@MnO_2_. (**K**) O1s XPS spectrum of MPDA-DOX@MnO_2_ (GSH). (**L**) DOX loading/encapsulation curves of MPDA-DOX.

Transmission electron microscopy (TEM) images were obtained to characterize the synthesis process and GSH-responsive degradation of MPDA-DOX@MnO_2_. The bare MPDA nanoparticles were roughly spherical with channel structure, and showed uniform size distribution with a diameter of 150 nm (Figure 1A). The channel of MnO_2_-DOX became blurry, owing to the occupation of DOX (Figure 1B). By coating MnO_2_, the core–shell structure was observed, and the diameter of MPDA-DOX@MnO_2_ was 220 nm (Figure 1C). By adding GSH, it was clearly found that the MnO_2_ layer disappeared, indicating the GSH-responsive degradation (Figure 1D). Further, the dynamic light scattering (DLS) and the potential were also measured. In Figure 1E, the hydrodynamic size of MPDA, MPDA-DOX, MPDA-DOX@MnO_2_ and MPDA-DOX@MnO_2_ (GSH) was 160, 167, 220 and 169 nm, and the corresponding zeta potential was −10.1, 8.5, −7.3 and 8.7 mV, in which the positive potential of MPDA-DOX can be attributed to the cationic DOX molecule (Supplementary Figure S1). It was observed that the morphology, size and potential of MPDA-DOX@MnO_2_ (GSH) were similar to those of MPDA-DOX, demonstrating the synthesis of MPDA-DOX@MnO_2_ and the degradation of MnO_2_.

By comparing the absorption peaks of different samples, a typical DOX peak at 488 nm was observed in DOX, MPDA-DOX, MPDA-DOX@MnO_2_ and MPDA-DOX@MnO_2_ (GSH), indicating the loading of DOX. Moreover, a broadened band at 400 nm belonged to MnO_2_ appeared in MPDA-DOX@MnO_2_ [46], but disappeared in that of MPDA-DOX@MnO_2_ (GSH), demonstrating the coating and degradation of MnO_2_ (Figure 1F). Moreover, in the FT-IR spectra of Figure 1G, the stretching vibration of O–H (3340 cm^−1^) and the stretching vibration of C=C (1630 cm^−1^) belonged to MPDA were observed, and the characteristic peak of DOX (1724 cm^−1^) was also found in the FT-IR spectrum of MPDA-DOX. By coating MnO_2_, the absorption peak at 504 cm^−1^ was assigned to the Mn–O bond [47], but disappeared with the presence of GSH. In addition, XPS spectra exhibited the peaks of C1s, N1s, O1s and Mn2p (Figure 1H). Further, Mn2p was divided into the two spin–orbit peaks of Mn2p_3/2_ (642.5 eV) and Mn2p_1/2_ (653.25 eV) (Figure 1I) [48]. By comparing the O1s spectra of different samples, the bonds of Mn–OH (527.82 eV) and Mn–O–Mn (529.07 eV) appeared in MPDA-DOX@MnO_2_ [49] (Figure 1J), but only the bonds of C–O (533.89 eV) and C=O (531.25 eV) were in MPDA-DOX@MnO_2_ (GSH) (Figure 1K) and MPDA-DOX (Supplementary Figure S2), confirming the degradation of MnO_2_.

In order to demonstrate the mesoporous structure of MPDA, the N_2_ adsorption-desorption isotherms and pore size were measured. The BET surface area of MPDA was 29 m^2^/g (Supplementary Figure S3A), and the pore size distribution ranged from 2.5 to 20 nm, in which the two main peaks of pore size were located at 3.4 and 10.8 nm (Supplementary Figure S3B). By varying the concentration of DOX, the optimal rates of loading and encapsulation were calculated to be 52% and 92% (Figure 1L; Supplementary Figure S4), in which the high loading/encapsulation efficiency was related to the mesoporous structure and negative surface potential of MPDA.

### Coating and GSH-responsive degradation of MnO_2_

**Figure 2.**
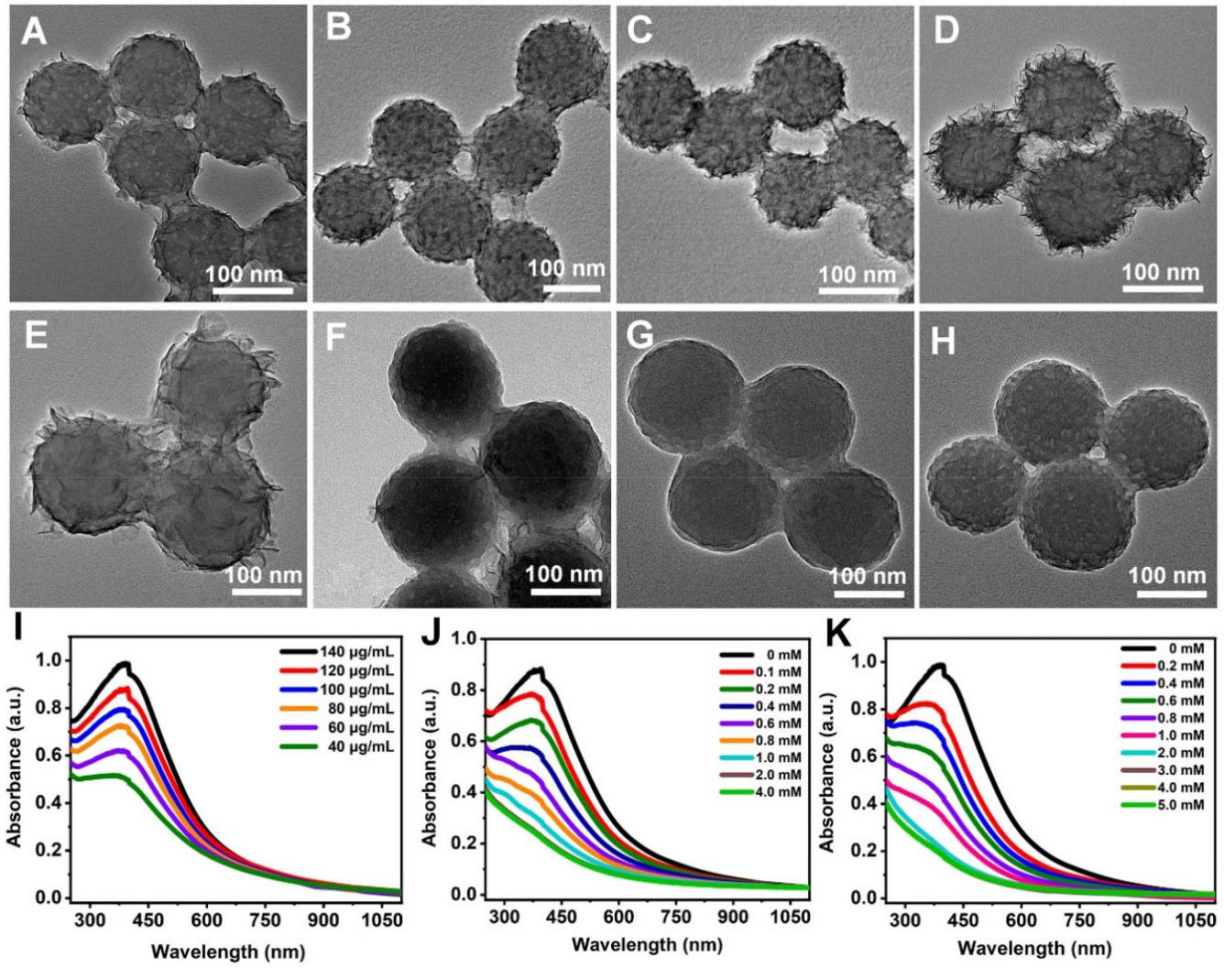
(**A–D**) TEM images of MPDA@MnO_2_ synthesized with KMnO_4_ at the concentrations of 60, 80, 100 and 140 μg/ml. (**E–H**) TEM images of MPDA@MnO_2_ synthesized with KMnO_4_ (120 μg/ml) with GSH at the concentrations of 0.4, 0.8, 1.0 and 2.0 mM. (**I**) UV–vis absorption spectra of MPDA@MnO_2_ synthesized with different KMnO_4_ concentrations. (**J**, **K**) UV–vis absorption spectra of MPDA@MnO_2_ synthesized with KMnO_4_ at the concentrations of 120 μg/ml and 140 μg/ml by changing GSH concentrations.

By changing KMnO_4_ concentration, MPDA@MnO_2_ nanoparticles with different thickness of MnO_2_ shell were prepared. As shown in Figures 1C and 2A–D and Supplementary Figure S5, when the concentration of KMnO_4_ increased from 40 to 140 μg/ml, the mesoporous structure of MPDA gradually disappeared, and the overall size of MPDA@MnO_2_ increased from 150 to 225 nm. The absorbance of MnO_2_ at 400 nm became larger by adding KMnO_4_ concentration (Figure 2I), indicating the increased thickness of MnO_2_ shell.

Fixing the GSH concentration (2 mM), the degradation behavior of MPDA@MnO_2_ with different thickness was investigated. As shown in Supplementary Figure S6, the MnO_2_ shell synthesized with a KMnO_4_ concentration below 120 μg/ml disappeared completely, but was still present, when the KMnO_4_ concentration was 140 μg/ml. By increasing GSH concentration from 0 to 2 mM, the MnO_2_ shell synthesized with KMnO_4_ at different concentration got thinner (Figure 2E–H; Supplementary Figures S7–S9). In addition, with the presence of GSH at different concentrations, the UV–vis absorption spectra exhibited that the absorbance of MnO_2_ gradually decreased, in which the complete degradation of MnO_2_ synthesized with KMnO_4_ at the concentrations of 40–140 μg/ml corresponded to a GSH concentration of 0.15, 0.4, 0.8, 1.0, 2.0 and 3.0 mM, respectively (Figure 2J and K; Supplementary Figure S10).

### GSH-responsive release of DOX

**Figure 3.**
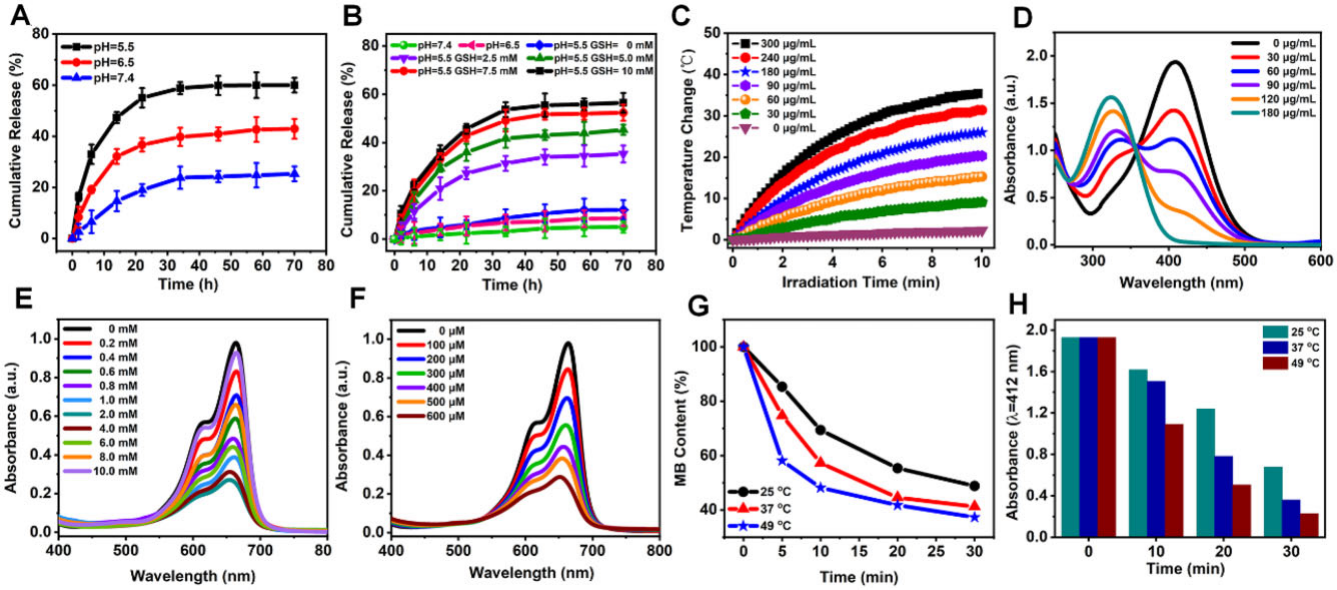
(**A**) DOX release curves of MPDA-DOX at different pH values. (**B**) DOX release curves of MPDA-DOX@MnO_2_ at different pH values and GSH concentrations. (**C**) Temperature variation curves of MPDA-DOX@MnO_2_. (**D**) Absorbance of DTNB incubated with MPDA@MnO_2_. (**E**, **F**) Absorbance of MB incubated with MPDA@MnO_2_ by varying GSH and H_2_O_2_ concentrations. (**G**) Absorbance curves of MB incubated with MPDA@MnO_2_ at different temperature. (**H**) Absorbance curves of TNB incubated with MPDA@MnO_2_ containing GSH at different temperature.

The release behavior of DOX in MPDA-DOX and MPDA-DOX@MnO_2_ was examined, respectively. when the pH of PBS was 7.4, 6.5 and 5.5, the cumulative release rates of DOX were 25.3%, 42.9% and 59.9% in MPDA-DOX (Figure 3A), but only 4.9%, 8.6% and 12.1% in MPDA-DOX@MnO_2_ (Figure 3B), in which the low release rates can be attributed to the protection of MnO_2_. In contrast, the DOX release of MPDA-DOX@MnO_2_ in PBS (pH = 5.5) was significantly accelerated by adding GSH concentration, in which the release rate reached 56.3%, when the GSH concentration was 10 mM (Figure 3B), which suggesting the GSH-responsive controllable release of DOX.

### Photothermal conversion, ·OH generation and GSH consumption

Under 808 nm laser irradiation, the temperature of MPDA-DOX@MnO_2_ increased by 25°C (Figure 3C; Supplementary Figure S11), and the temperature change was nearly constant by repeating on/off laser (Supplementary Figure S12). Moreover, the photothermal conversion efficiency (η) was calculated was 40.1% (Supplementary Figure S13), indicating the good photothermal conversion and stability of MPDA-DOX@MnO_2_. In TME, the overexpressed GSH can scavenge the produced ·OH to reduce CDT performance, and MnO_2_ can be a candidate as an effective depleting agent of GSH, in which DTNB is used as a probe to detect the depletion of GSH. As shown in Figure 3D, by increasing the concentration of MPDA@MnO_2_, the absorbance of TNB at 412 nm significantly decreased, indicating the good GSH depletion performance of MnO_2_. Moreover, it was found that the absorbance of MB first decreased, and then increased by adding GSH concentration to above 2 mM (Figure 3E), demonstrating the ·OH elimination of excess GSH.

With the increase of exogenous H_2_O_2_, the ·OH production of MPDA@MnO_2_ was measured. In Figure 3F, when the concentration of H_2_O_2_ was 600 μM, the absorbance of MB greatly decreased by 74.4%, Moreover, it was found that the ·OH production of MPDA@MnO_2_ can be enhanced by improving incubation temperature. It was firstly confirmed that single MB showed good temperature stability (Supplementary Figure S14). When the incubation temperature was 25, 37 and 49°C, the absorbance of MB incubated with MPDA@MnO_2_ decreased by 52%, 59% and 63% (Figure 3G; Supplementary Figures S15–S17), respectively. By comparing the ·OH production MPDA@MnO_2_ with different shell thickness, it was observed that the thicker MnO_2_ shell consumed more MB (Supplementary Figures S18–S20), which was relevant with the different Mn^2+^ amounts. In addition, the GSH depletion of MPDA@MnO_2_ can also be accelerated by improving incubation temperature. As shown in Figure 3H and Supplementary Figures S21–S23, the absorbance of TNB at 412 nm decreased by 64.8%, 81.3% and 88.2% under the incubation temperature of 25, 37 and 49°C. All the above results indicated that the photothermal performance of MPDA can enhance the ·OH production.

### Biocompatibility and *in vitro* CDT/PTT/chemotherapy

**Figure 4.**
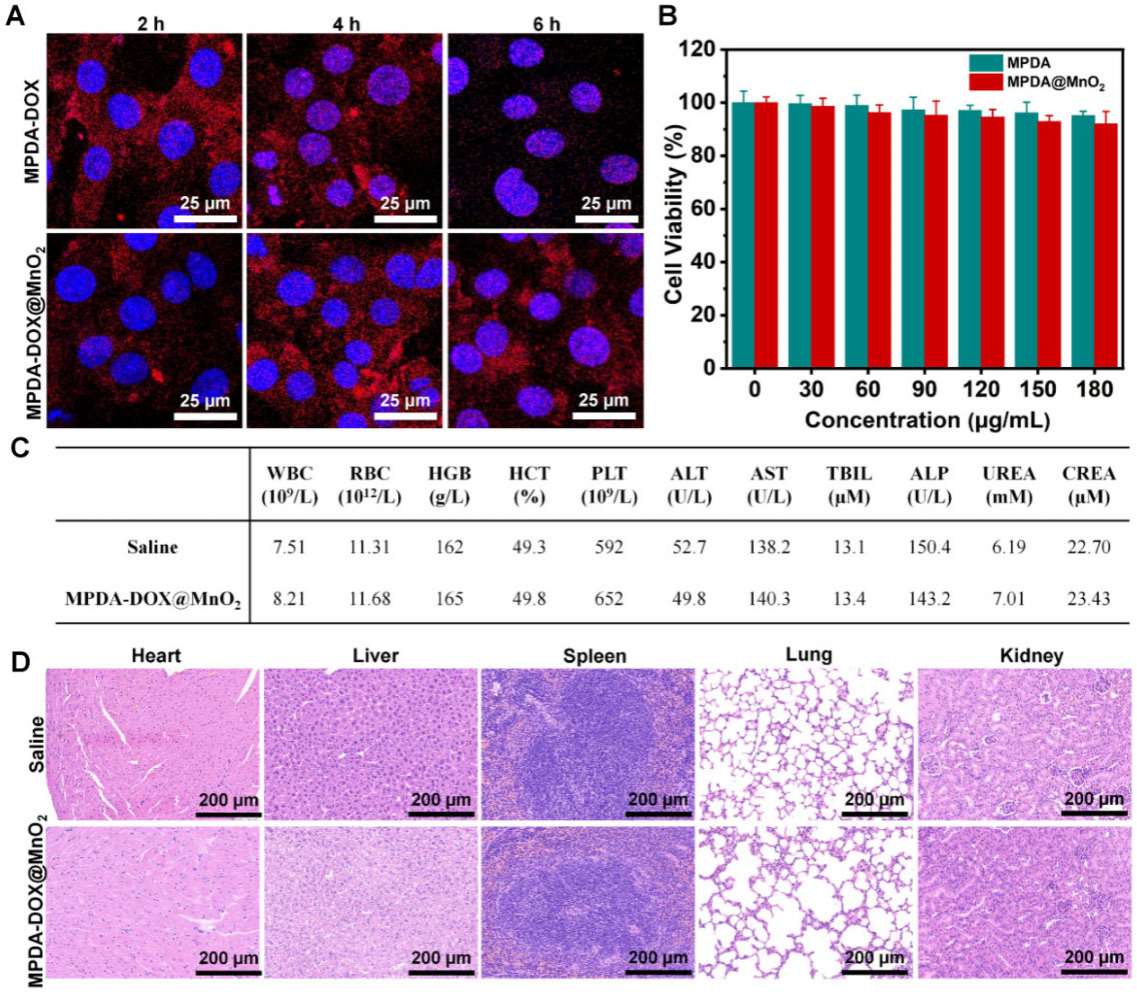
(**A**) LSCM images of MPDA-DOX and MPDA-DOX@MnO_2_. (**B**) Cell viabilities of different samples. (**C**) Blood routine and biochemical indexes. (**D**) H&E staining images.

In order to demonstrate the controllable release capability of DOX, the cellular uptake behavior of MPDA-DOX and MPDA-DOX@MnO_2_ was firstly compared. In Figure 4A, the laser scanning confocal microscopy (LSCM) images of MPDA-DOX can rapidly enter cells, and release in the nucleus. However, the cellular uptake of MPDA-DOX@MnO_2_ was slowly, and only a portion of DOX entered into the nucleus after 6 h incubation, which suggested that GSH-responsive MnO_2_ degradation can modulate the release behavior of DOX. Then, the cytotoxicity of MPDA and MPDA@MnO_2_ was determined. After 24 h incubation, the viability incubated with MPDA and MPDA@MnO_2_ was still above 95% even at the highest concentration of 180 µg/ml (Figure 4B). By comparing the mice injected with saline and MPDA-DOX@MnO_2_, no significant difference was found in the indexes of blood routine and biochemistry (Figure 4C). Furthermore, using H&E staining, no significant necrosis and fibrosis were observed in the organs (Figure 4D). All the results suggested that the MPDA-DOX@MnO_2_ nanoprobe possessed good biocompatibility.

**Figure 5.**
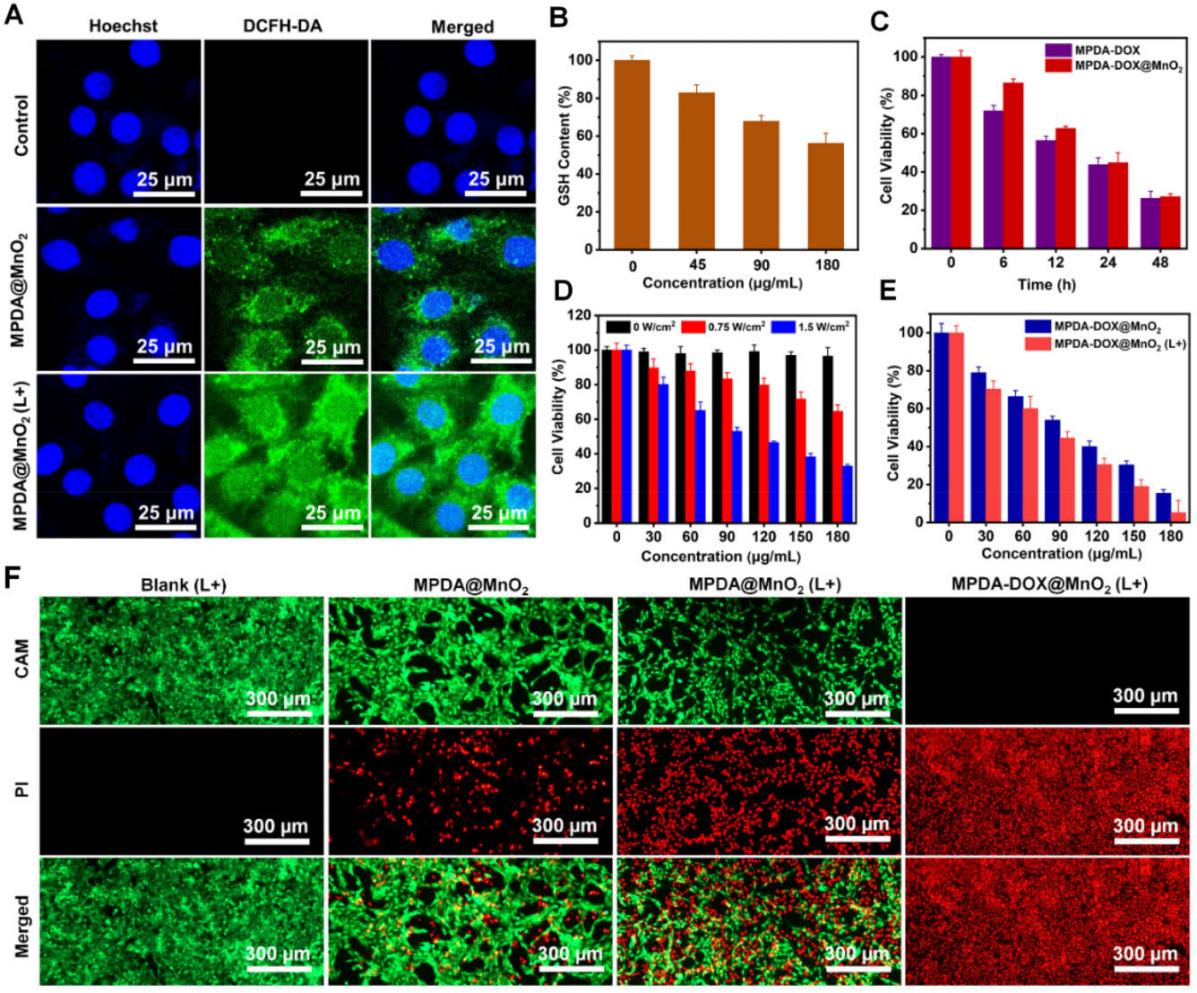
(**A**) LSCM images of different samples by measuring ·OH production. (**B**) Intracellular GSH content incubated with MPDA-DOX@MnO_2_. (**C**) *In vitro* chemotherapy. (**D**) *In vitro* PTT. (**E**) *In vitro* CDT/PTT/chemotherapy. (**F**) CAM/PI staining images of different samples (L+: 808 nm laser, 1.0 W/cm^2^, 5 min).

To detect intracellular ·OH production, DCFH-DA was applied as a fluorescent indicator. In Figure 5A, green fluorescence was clearly observed in the sample of MPDA@MnO_2_, and the fluorescence got stronger after laser irradiation, indicating the photothermal-enhanced ·OH production. Moreover, the intracellular GSH content can reduce to 53%, when the concentration of MPDA@MnO_2_ was 180 μg/ml (Figure 5B), demonstrating the effective depletion of intracellular GSH level.

The performances of *in vitro* chemotherapy, PTT and CDT were evaluated, respectively. In Figure 5C, the chemotherapy performance of MPDA-DOX@MnO_2_ was worse than MPDA-DOX with the incubation time of below 12 h, but the viability was almost equivalent after 24 h incubation owing to the slow-release effect of MPDA-DOX@MnO_2_, in which the viability can reduce to 26%. Under the laser irradiation with different power density (0.75 and 1.50 W/cm^2^), the single PTT performance of MPDA@MnO_2_ decreased the viability to 63% and 31% (Figure 5D). In addition, by adding H_2_O_2_ at the concentrations of 50 and 100 μM, the single CDT performance of MPDA@MnO_2_ decrease the cell viability to 76% and 57%, respectively (Supplementary Figure S24).

Further, the combination therapy performance of MPDA-DOX@MnO_2_ was measured. As shown in Figure 5E, the viability decreased to 15% by CDT/chemotherapy, but can decrease to 6% by CDT/PTT/chemotherapy. As shown in Figure 5F, the live/dead cells staining indicated that only weak red fluorescence was observed in the group of MPDA@MnO_2_, but the red fluorescence was strong under laser irradiation. Importantly, in the group of MPDA-DOX@MnO_2_ (L+), the green fluorescence was nearly disappeared, which was also consistent with the MTT assay. All the above results demonstrated the good *in vitro* CDT/PTT/chemotherapy performances of MPDA-DOX@MnO_2_.

### *In vivo* CDT/PTT/chemotherapy

**Figure 6.**
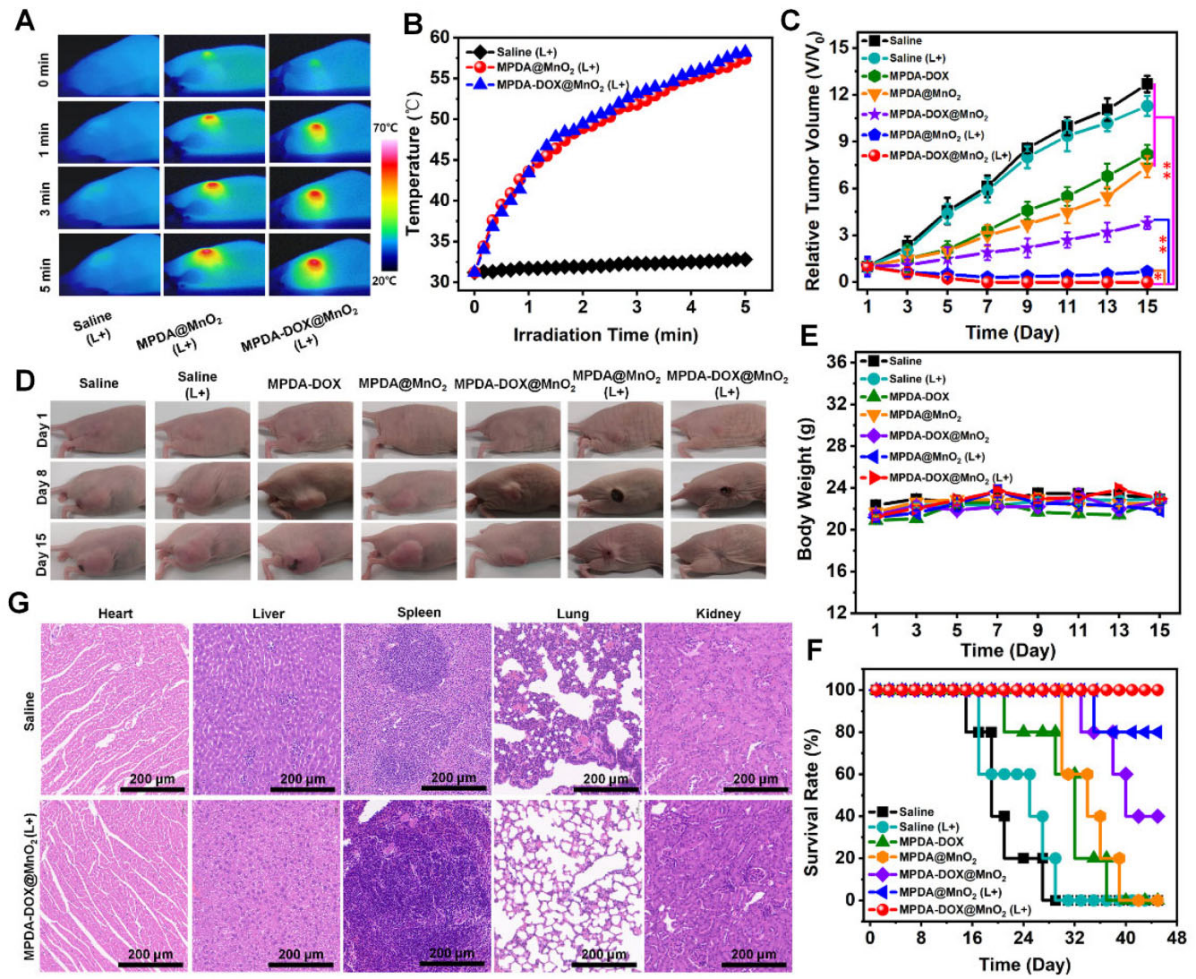
(**A**) Thermal imaging. (**B**) Temperature curves. (**C**) Tumor volume curves (**P < 0.01, *P < 0.05). (**D**) Body weight curves. (**E**) Survival rate curves. (**F**) Real photographs. (**G**) H&E staining images.

By building MDA-MB-435 tumor-bearing nude mice, the *in vivo* anti-cancer efficacy of MPDA-DOX@MnO_2_ was evaluated. After the tumor sites injected with MPDA@MnO_2_ and MPDA-DOX@MnO_2_ were irradiated, the temperature increased to 56.4°C and 57.3°C, but the temperature was almost constant in the control group, indicating the good tumor accumulation (Figure 6A and B). In 14 days, the tumors in Saline, Saline (L+), MPDA-DOX, MPDA@MnO_2_, and MPDA-DOX@MnO_2_ grew by 12.7, 11.3, 8.2, 7.4 and 3.8 times. In the group of MPDA@MnO_2_ (L+), the tumor size firstly decreased and then increased to ∼0.67 times. However, in the group of MPDA-DOX@MnO_2_ (L+), the tumors completely disappeared (Figure 6C and D), demonstrating the good CDT/PTT/chemotherapy. Compared with the mice in other groups, no body weight loss was found (Figure 6E). Importantly, in the group of MPDA-DOX@MnO_2_ (L+), the survival rate was 100% (Figure 6F). By comparing the H&E staining of different organs, no metastasis was observed in the group of MPDA-DOX@MnO_2_ (L+), but tiny liver metastasis appeared in the group of Saline (Figure 6G). All the results demonstrated the safety and high efficacy of CDT/PTT/chemotherapy.

## Conclusion

In summary, using MnO_2_ as gatekeeper, the TME-responsive MPDA-DOX@MnO_2_ nanoplatform was constructed for modulating DOX release, by which the non-specific leakage of DOX was avoided, and the synergistic PTT, CDT, and chemotherapy were achieved. The results indicated MnO_2_ shell protected DOX from leakage in normal physiological environment, while caused the DOX release in TME, owing to the GSH-responsive MnO_2_ degradation. By adding GSH concentration, the DOX release of MPDA-DOX@MnO_2_ (pH = 5.5) increased from 12.1% to 56.3%. Moreover, the MnO_2_ shell also acted as a GSH depleting agent, significantly reducing the ·OH clearance. Using the endogenous H_2_O_2_ and the photothermal conversion of MPDA, the ·OH generation was promoted. Using the synergistic CDT/PTT/chemotherapy of MPDA-DOX@MnO_2_, the cell viability decreased to 6%, and the tumors were eliminated. Thus, this work designed TME-responsive delivery nanoplatform, and achieved the synergistic CDT/PTT/chemotherapy with high-safety and high-efficacy against tumors.

## Supplementary Material

rbad087_Supplementary_Data
