# Supplementary material for: Mesoporous polydopamine delivery system for intelligent drug release and photothermal-enhanced chemodynamic therapy using MnO2 as gatekeeper
Source: Regen Biomater. 2023 Sep 22;10:rbad087. doi: 10.1093/rb/rbad087 (PMC10627289; doi:10.1093/rb/rbad087)
Supplement: rbad087_Supplementary_Data [file rbad087_supplementary_data.docx]

**Support Information**

**Mesoporous polydopamine delivery system for** **intelligent drug release and photothermal-enhanced chemodynamic therapy using MnO_2_ as gatekeeper**

Zhaoyang Wang^#^, Zekai Li^#^, Yuehua Shi, and Leyong Zeng*

*Key Laboratory of Medicinal Chemistry and Molecular Diagnosis of the Ministry of Education, Chemical Biology Key Laboratory of Hebei Province, State Key Laboratory of New Pharmaceutical Preparations and Excipients, College of Chemistry and Materials Science, Hebei University, Baoding, 071002, P.R. China.*

*Corresponding author. E-mail: zengly@hbu.cn

^#^These authors contributed equally to this work.

**Figure S**1. Zeta potential of different samples.





**Figure S**2. O1s XPS spectrum of MPDA-DOX.


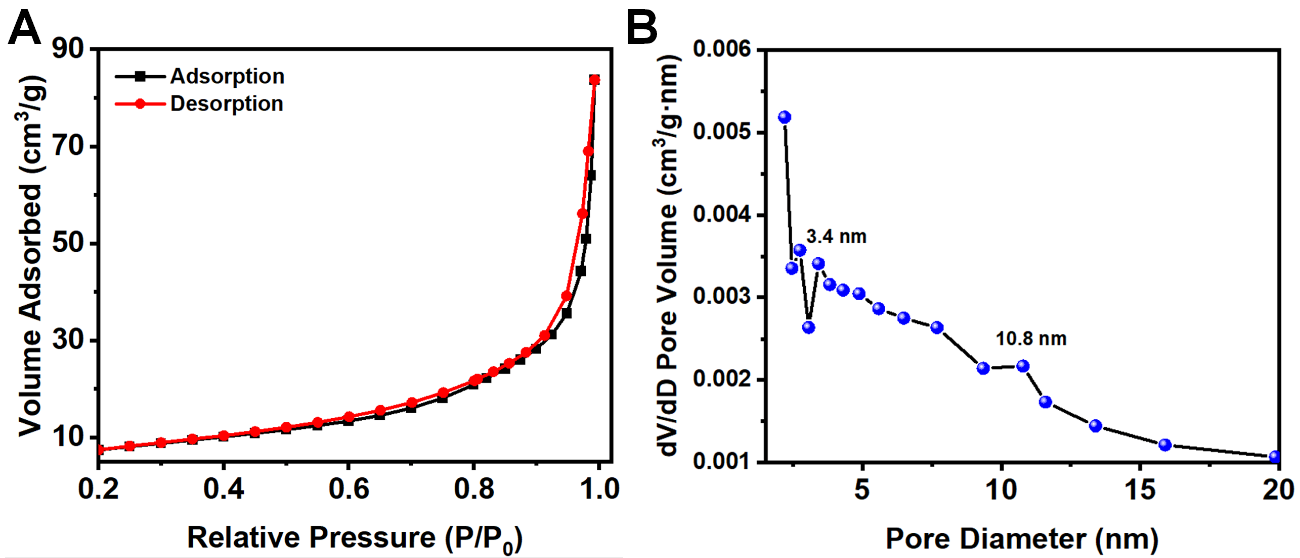


**Figure S**3. (A) N_2_ adsorption-desorption isotherms of MPDA. (B) Pore size distribution of MPDA.





**Figure S**4. Standard curve of DOX.


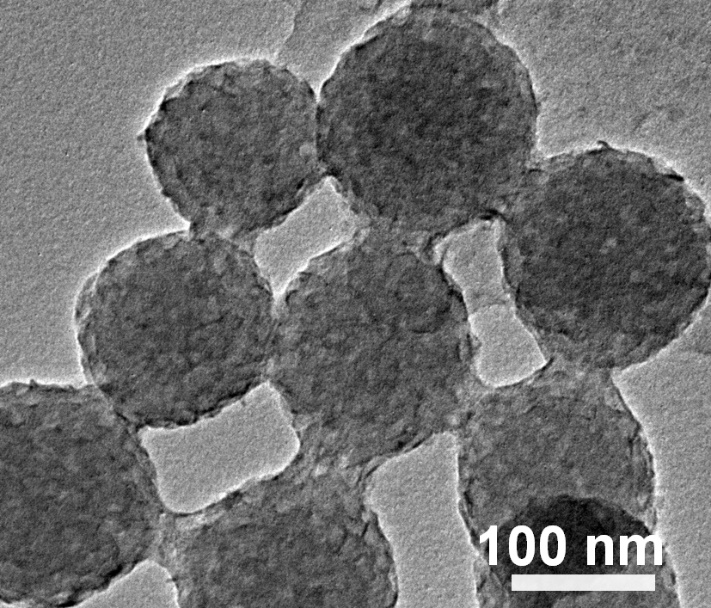


**Figure S**5. TEM images of MPDA@MnO_2_ synthesized with KMnO_4_ at the concentration of 40 μg/mL.


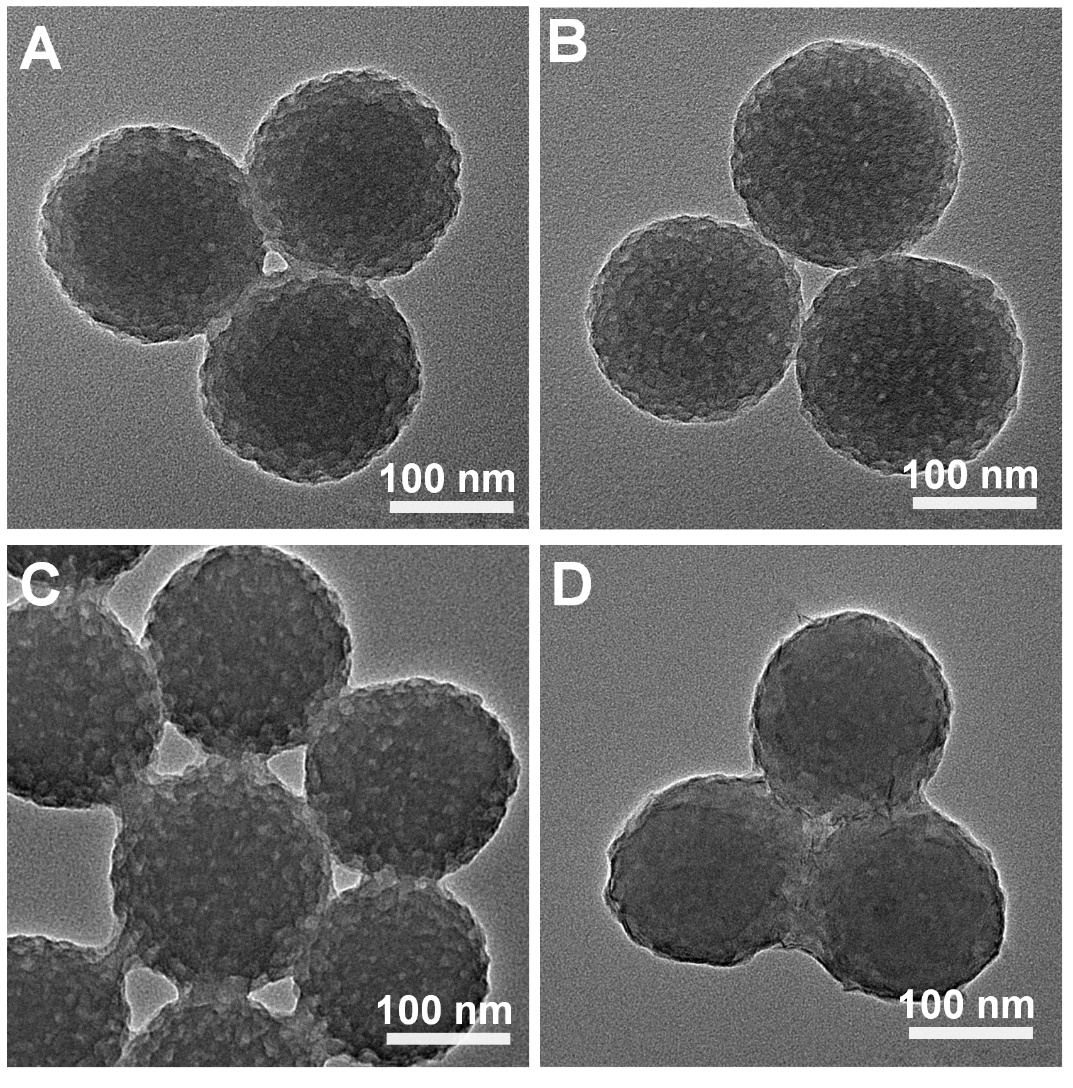


**Figure S**6. TEM images of MPDA@MnO_2_ synthesized with KMnO_4_ at different concentrations by adding GSH (2 mM). (A) 80 μg/mL. (B) 100 μg/mL. (C) 120 μg/mL. (D) 140 μg/mL.


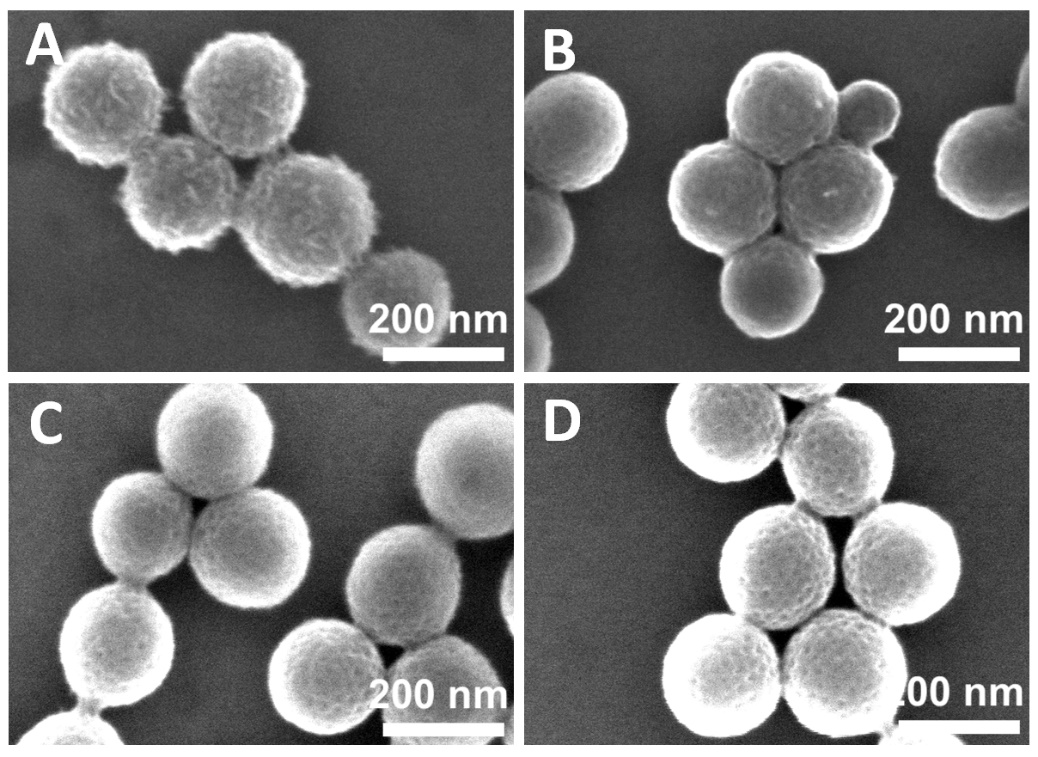


**Figure S**7. SEM images of MPDA@MnO_2_ synthesized with KMnO_4_ (100 μg/mL) by adding GSH at different concentrations. (A) 0 mM. (B) 0.6 mM. (C) 1.0 mM. (D) 2.0 mM.


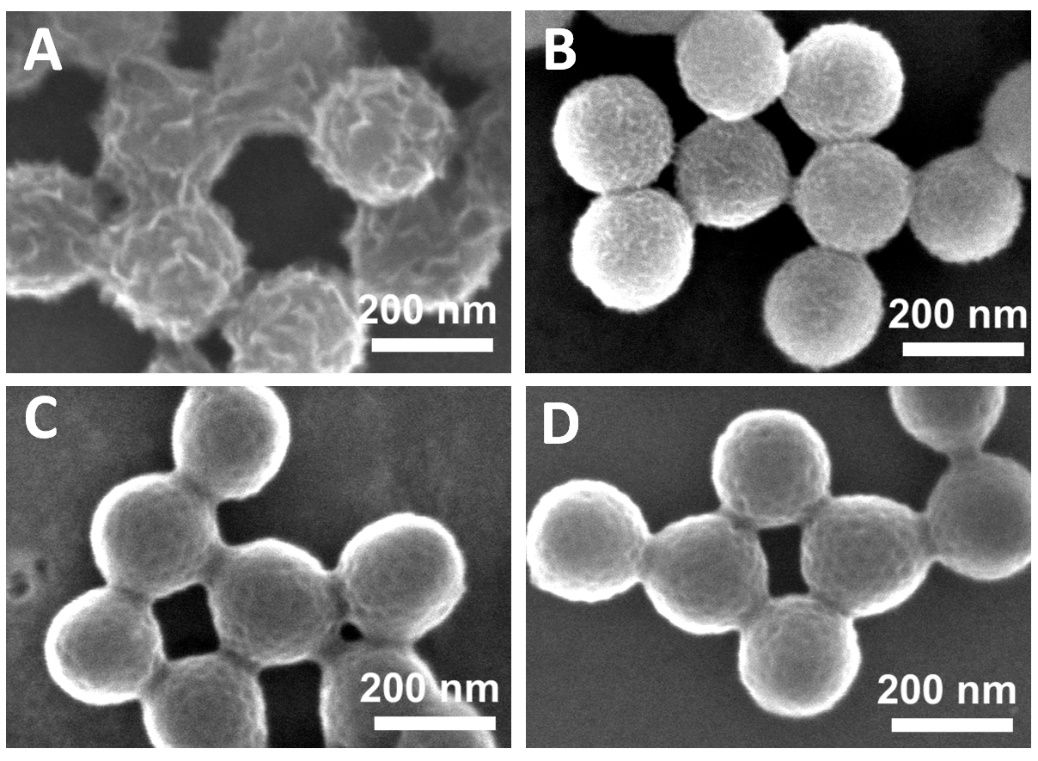


**Figure S**8. SEM images of MPDA@MnO_2_ synthesized with KMnO_4_ (120 μg/mL) by adding GSH at different concentrations. (A) 0 mM. (B) 0.6 mM. (C) 1.0 mM. (D) 2.0 mM.


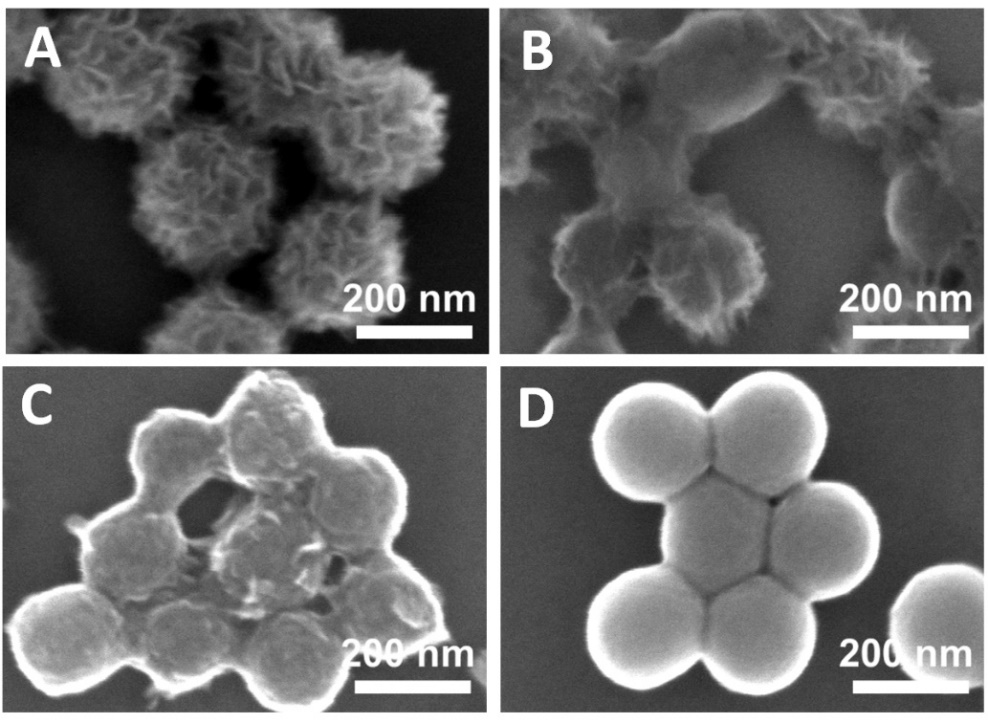


**Figure S**9. SEM images of MPDA@MnO_2_ synthesized with KMnO_4_ (140 μg/mL) by adding GSH at different concentrations. (A) 0 mM. (B) 0.6 mM. (C) 1.0 mM. (D) 2.0 mM.


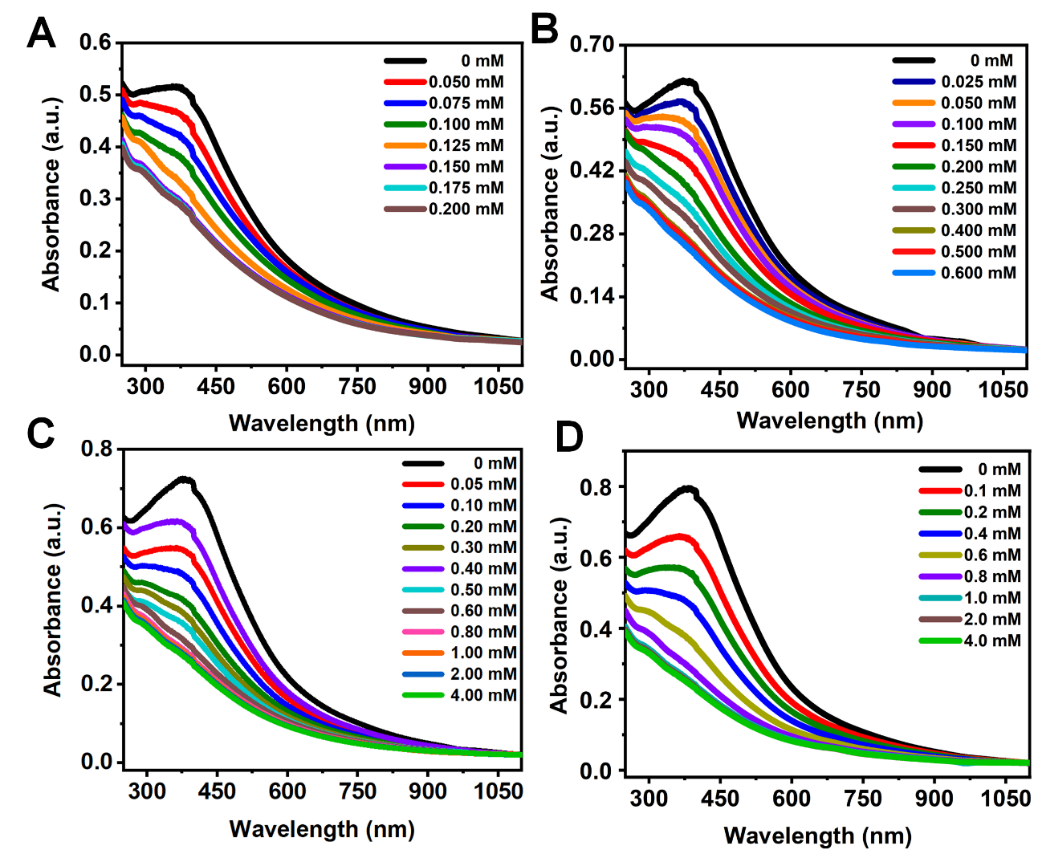


**Figure S**10. UV-vis absorption spectra of MPDA@MnO_2_ synthesized with KMnO_4_ at different concentrations by changing GSH concentrations. (A) 40 μg/mL. (B) 60 μg/mL. (C) 80 μg/mL. (D) 100 μg/mL.





**Figure S**11. Temperature variation curves of MPDA-DOX@MnO_2_ by changing power densities of laser.





**Figure S**12. Recycling heating curves of MPDA-DOX@MnO_2_ with laser (1.0 W/cm^2^).





**Figure S**13. Heating/cooling and fitting curves of MPDA-DOX@MnO_2_.


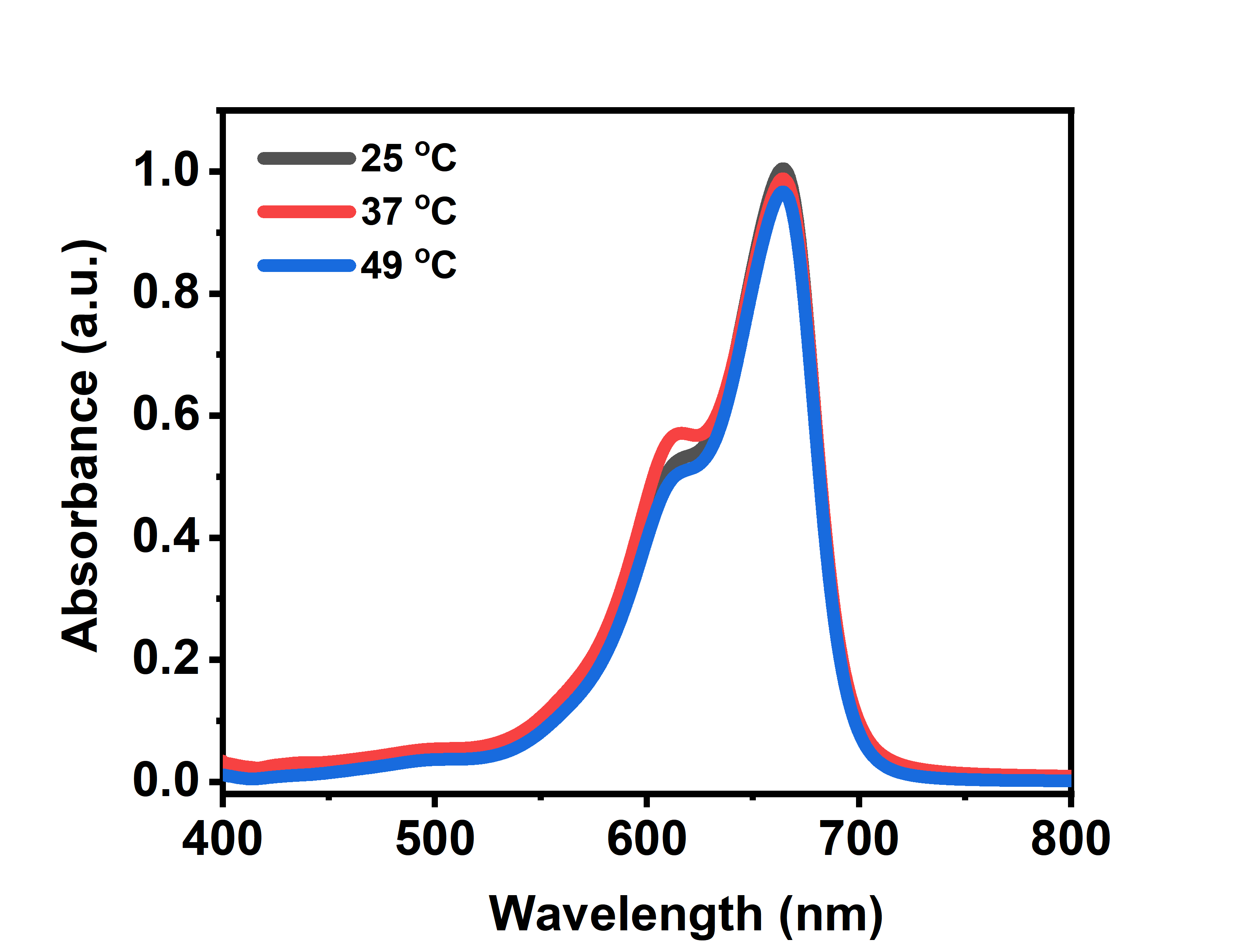


**Figure S**14. UV-vis absorption spectra of single MB at different temperature.





**Figure S**15. UV-vis absorption spectra of MB incubated with MPDA@MnO_2_ at 25 ℃.





**Figure S**16. UV-vis absorption spectra of MB incubated with MPDA@MnO_2_ at 37 ℃.





**Figure S**17. UV-vis absorption spectra of MB incubated with MPDA@MnO_2_ at 49 ℃.





**Figure S**18. UV-vis absorption spectra of MB incubated with MPDA@MnO_2_ synthesized with a KMnO_4_ concentration of 80 μg/mL.





**Figure S**19. UV-vis absorption spectra of MB incubated with MPDA@MnO_2_ synthesized with a KMnO_4_ concentration of 100 μg/mL.





**Figure S**20. UV-vis absorption spectra of MB incubated with MPDA@MnO_2_ synthesized with a KMnO_4_ concentration of 140 μg/mL.


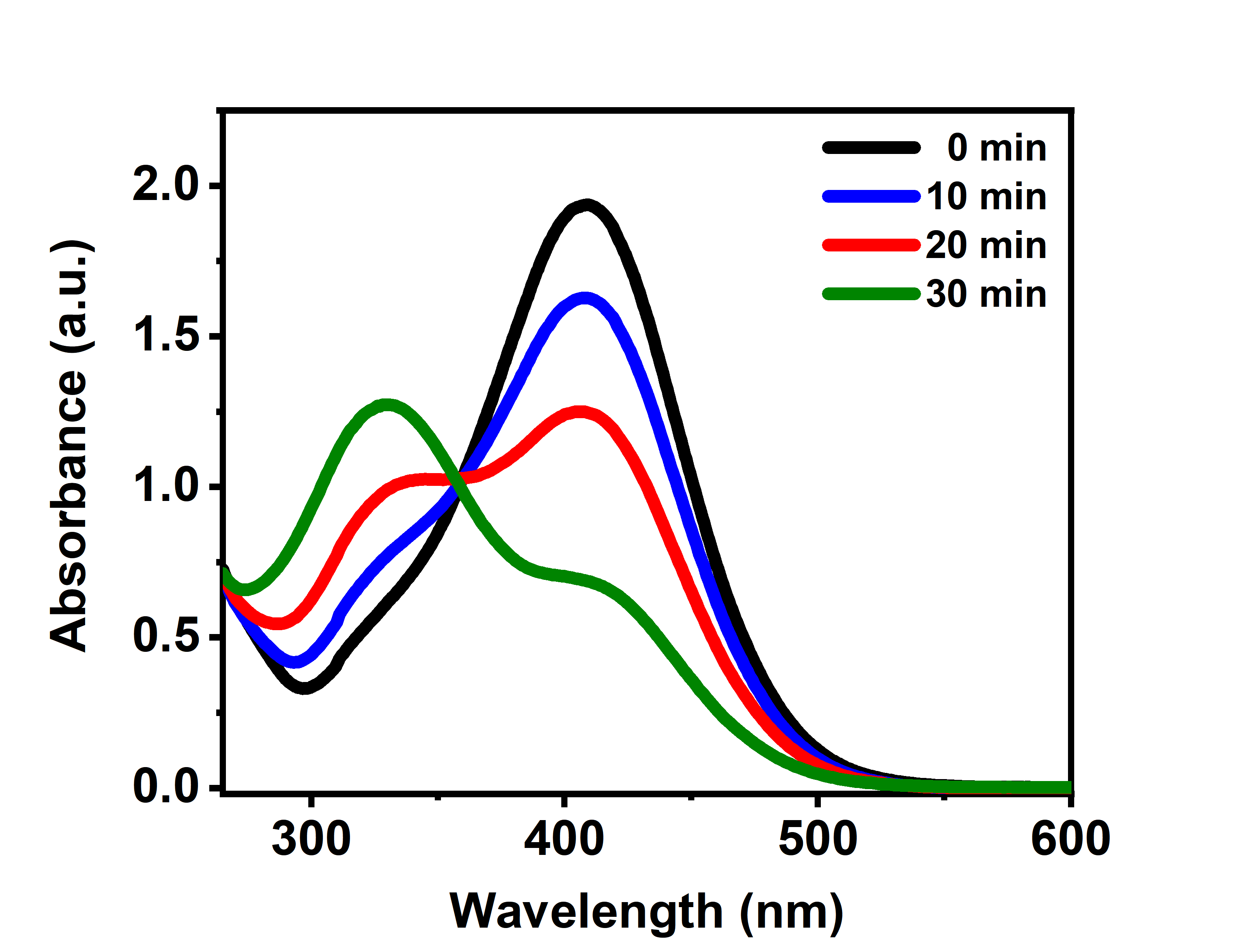


**Figure S**21. UV-vis absorption spectra of TNB incubated with MPDA@MnO_2_ at 25 ℃.


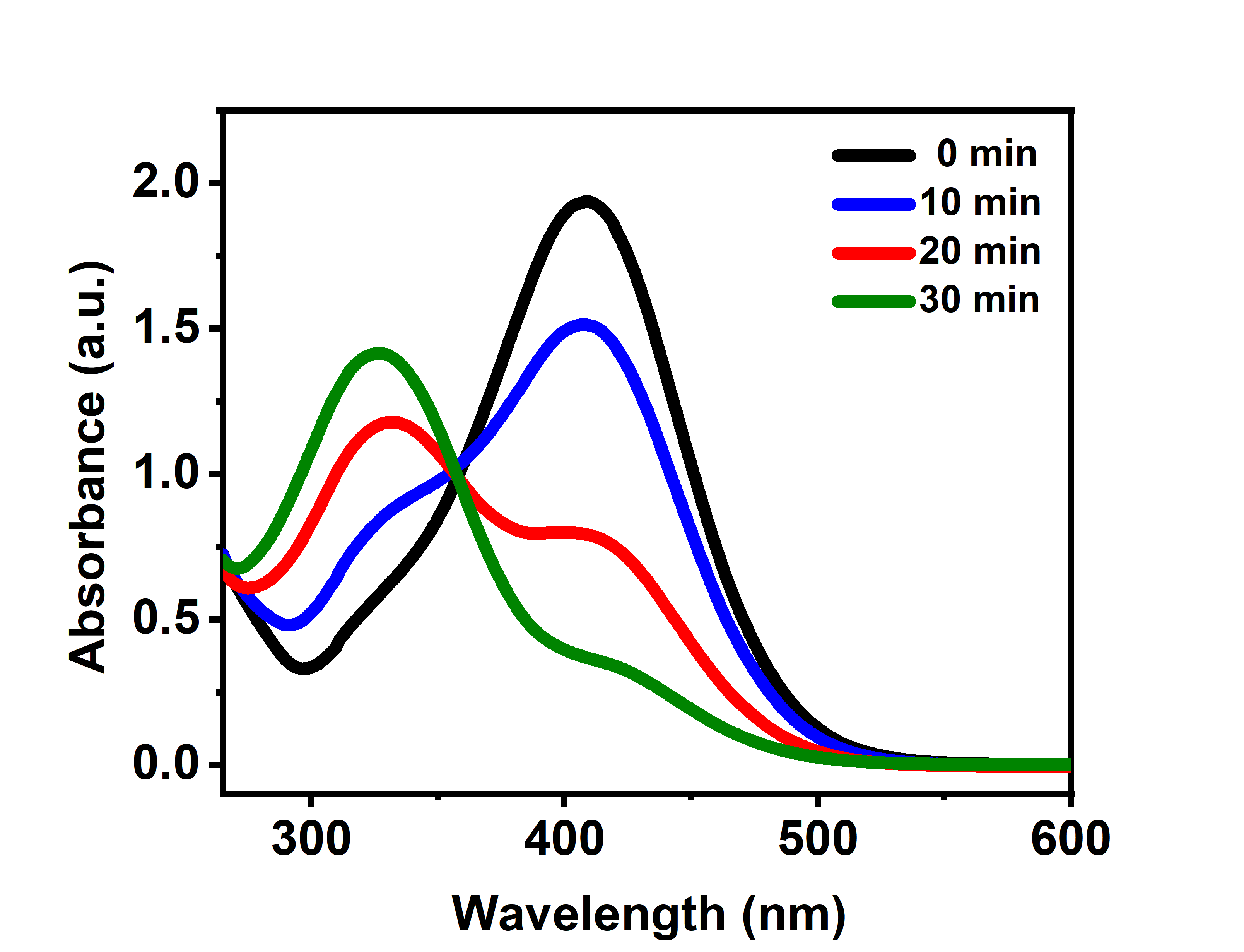


**Figure S**22. UV-vis absorption spectra of TNB incubated with MPDA@MnO_2_ at 37 ℃.


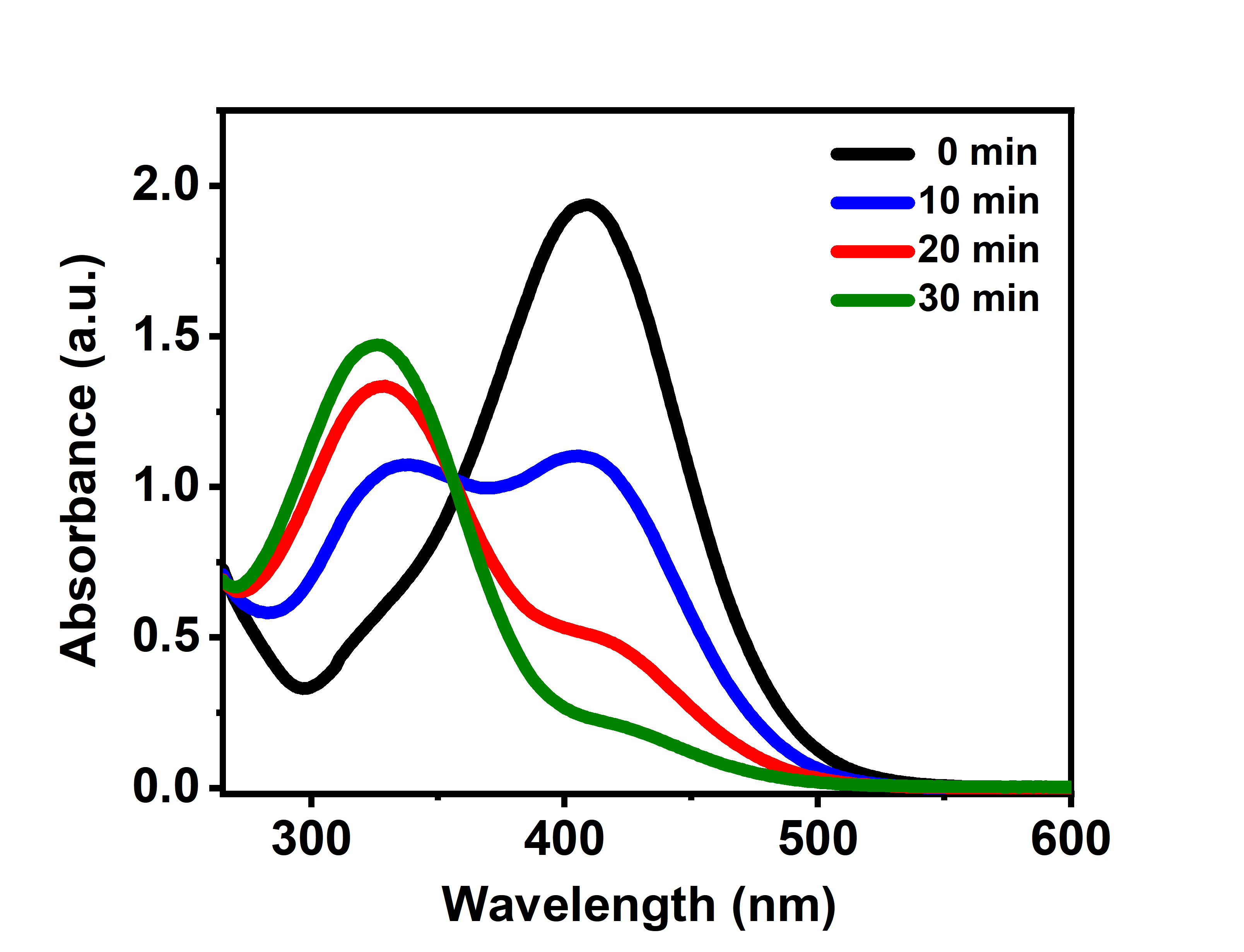


**Figure S**23. UV-vis absorption spectra of TNB incubated with MPDA@MnO_2_ at 49 ℃.





**Figure S**24. Cell viabilities of MDA-MB-435 cells incubated with different samples. I) Blank; Ⅱ) H_2_O_2_ (100 μM); Ⅲ) MPDA@MnO_2_; Ⅳ) MPDA@MnO_2_ containing H_2_O_2_ (50 μM); Ⅴ) MPDA@MnO_2_ containing H_2_O_2_ (100 μM).
